# PPAR*γ*, PTEN, and the Fight against Cancer

**DOI:** 10.1155/2008/932632

**Published:** 2008-12-14

**Authors:** Rosemary E. Teresi, Kristin A. Waite

**Affiliations:** ^1^Genomic Medicine Institute, Cleveland Clinic Foundation, Cleveland, OH 44195, USA; ^2^Lerner Research Institute, Cleveland Clinic Foundation, Cleveland, OH 44195, USA; ^3^Taussig Cancer Institute, Cleveland Clinic Foundation, Cleveland, OH 44195, USA

## Abstract

Peroxisome proliferator-activated receptor gamma (PPAR*γ*) is a ligand-activated transcription factor, which belongs to the family of nuclear hormone receptors. Recent in vitro studies have shown that PPAR*γ* can regulate the transcription of *phosphatase and tensin homolog on chromosome* *ten* (*PTEN*), a known tumor suppressor. *PTEN* is a susceptibility gene for a number of disorders, including breast and thyroid cancer. Activation of PPAR*γ* through agonists increases functional PTEN protein levels that subsequently induces apoptosis and inhibits cellular growth, which suggests that PPAR*γ* may be a tumor suppressor. Indeed, several in vivo studies have demonstrated that genetic alterations of PPAR*γ* can promote tumor progression. These results are supported by observations of the beneficial effects of PPAR*γ* agonists in the in vivo cancer setting. These studies signify the importance of PPAR*γ* and *PTEN*'s interaction in cancer prevention.

## 1. INTRODUCTION

### 1.1. PPAR*γ*

Peroxisome proliferator-activated receptor gamma (PPAR*γ*) is a ligand-activated transcription factor, belonging to the nuclear hormone receptor family, whose ligand-binding domain is located at the carboxy-terminus. There are several known natural and synthetic PPAR*γ* agonists with 15-deoxy-delta 12, 14-prostaglandin-J2 (15d-PG-J2) being the most notable natural PPAR*γ* agonist. Additionally, linoleic, linolenic, and arachidonic acids are other commonly recognized natural agonists. Synthetic PPAR*γ* agonists, such as the thiazolidinediones (TZDs), are some of the most commonly prescribed medications for the treatment of type II diabetes mellitus. The four commercially recognized TZDs are ciglitazone (Alexis), pioglitazone (Actos), rosiglitazone (Avandia), and troglitazone (Rezulin).

After ligand-activation, PPAR*γ* forms a heterodimer complex with retinoic acid receptor (RXR). This PPAR*γ*/RXR complex subsequently translocates to the nucleus and binds to a peroxisome proliferator response element (PPRE) within a target gene thereby initiating transcription. The primary, and most studied, targets of PPAR*γ* are involved in metabolic pathways and adipocyte differentiation. However, in recent years it has been suggested that PPAR*γ* has a role in cancer development. Indeed, initial studies demonstrated alterations of cellular differentiation, indicative of apoptosis in a breast cancer setting, after PPAR*γ* agonist stimulation. This indicates that PPAR*γ* and its agonists may play an important role in cancer development, prevention, and treatment.

In 1998, Mueller et al. performed one of the first PPAR*γ* agonist studies in a cancer setting [1]. They demonstrated that both 15d-PG-J2 and rosiglitazone (Rosi) could induce changes in epithelial gene expression associated with a more differentiated, less malignant state. Moreover, they described a reduction in the overall growth rate of breast cancer cells when treated with a PPAR*γ* agonist. These data suggest that PPAR*γ* can contribute to the prevention of breast cancer development and its agonists may be a novel therapy for cancer treatment [1]. These results stimulated further studies investigating PPAR*γ*-mediated tumor suppression. One protein, that may play a role in PPAR*γ*-mediated tumor suppression, is phosphatase and tensin homolog on chromosome ten (PTEN), which has an established role in breast cancer development. Interestingly, Mueller et al. characterization of breast cancer cells after PPAR*γ* activation demonstrated a striking resemblance to cells with active PTEN expression [1]. Taken together, these results suggested that PTEN and PPAR*γ*, together, may modulate breast cancer progression.

### 1.2. PTEN

In 1995, *PTEN* was identified as the susceptibility gene for Cowden syndrome (CS), which is characterized by breast, thyroid, and endometrial carcinoma as well as macrocephaly [2–8]. Patients diagnosed with CS have a 25–50% lifetime risk of developing female breast cancer, compared to the general population risk of ~13% [9, 10] Additionally, patients have ~10% lifetime risk of developing thyroid cancer, compared to <1% in the general population and have a ~5–10% lifetime risk of endometrial cancer compared to ~2–4% in the general population [9, 11]. Since its identification, research has detected a *PTEN* mutation in 85% of CS patients [11]. Furthermore, somatic alterations in *PTEN*, whether by genetic or epigenetic mechanisms, play some role in the pathogenesis of a broad range of solid tumors, including sporadic carcinomas of the breast, thyroid, endometrium, and colon.

**Figure 1:**
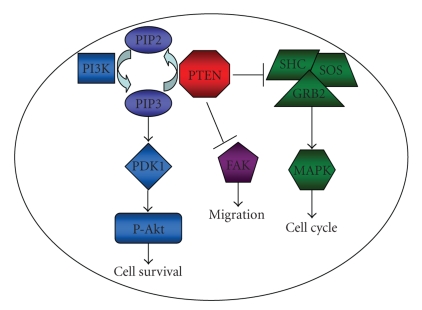
PTEN protein signaling pathways. PTEN’s lipid phosphatase activity dephosphorylates PIP3 to PIP2 inhibiting PDK1-mediated Akt phosphorylation and downregulating Akt-mediated cell survival. PTEN’s protein phosphatase activity inhibits the phosphorylation of FAK to prevent cell migration. PTEN’s protein phosphatase activity also dephosphorylates the SHC/SOS/GRB2 complex resulting in the decreased phosphorylation of MAPK and inhibition of the cell cycle.

*PTEN*’s protein, PTEN, is a unique phosphatase that has the ability to dephosphorylate both proteins and lipids (Figure 1) [4]. Its lipid phosphatase activity functions as a negative regulator of Akt phosphorylation (P-Akt). PTEN dephosphorylates phosphatidylinositol-3,4,5-triphosphate (PIP3) at the D3 position generating phosphatidylinositol 4,5-biphosphate (PIP2), decreasing cellular PIP3 levels. Since PIP3 is required for Akt phosphorylation, active PTEN leads to a decrease in the levels of P-Akt and consequently a decrease in Akt-mediated proliferation pathways. PTEN’s protein phosphatase activity has been shown to inhibit the SHC/SOS/GRB2 and mitogen-activated protein kinase (MAPK) pathways. The dephosphorylation of SHC by PTEN indirectly decreases the phosphorylated form of MAPK levels, reducing MAPK’s activity. Additionally, PTEN’s protein phosphatase activity upregulates p27 with a concomitant downregulation of cyclin D1 which coordinates G1 arrest [12]. By regulating these key-signaling pathways, PTEN downregulates cell division and upregulates apoptosis. Additionally, PTEN’s protein phosphatase activity has been shown to dephosphorylate focal adhesion kinase (FAK), which inhibits cell spreading and migration [4].

**Figure 2:**
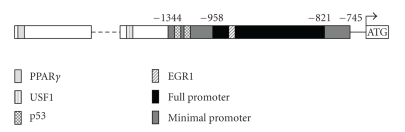
*PTEN* promoter and its transcription factors. *PTEN*’s full-length promoter lies between −1344 and −745 (gray bar), while the minimal promoter lies between −958 and −821 (black bar). Four transcription factors are known to directly bind upstream of PTEN: PPAR*γ* (solid gray bar), USF1 (stripped gray bar), p53 (dotted gray bar), and EGR1 (dashed gray bar).

Transcriptional regulation of *PTEN* is only beginning to be elucidated. To date, analysis of *PTEN’*s promoter suggests that there are at least eight regulatory factors that modulate *PTEN*’s transcription (Figure 2). In 2001, Stambolic et al. identified a functional p53 binding site, located at nucleotide positions −1190 to −1157 in *PTEN*’s promoter, which was required for *PTEN’s* upregulation [13]. Additionally, early growth response-1 (Egr-1) has been shown to bind to the *PTEN* promoter at −947 to −939 and induce PTEN expression [14]. Recently, our laboratory has identified a USF1 binding site ~2 kb (−2237 and −2058) upstream of the ATG site [15]. CBF-1, Sp1, and c-Jun have also recently been suggested as *PTEN* transcription factors [11, 16–18]. The majority of *PTEN* promoter analyses have been focused on transcription factors that increase PTEN levels. However, recently, suppression of *PTEN* gene expression has been shown by the tumor necrosis factor-alpha/nuclear factor-kappa B (NF-*κ*B) [19], however the precise mechanism of this inhibition remains unclear.

### 1.3. PPAR*γ* and PTEN in vitro

In 2001, Patel et al. first showed that PPAR*γ* can be a *PTEN* transcription factor [20]. They observed that Rosi induced PTEN protein expression in both MCF-7 breast and CoCa2 colon cancer cell lines. In addition to the increase in PTEN expression, they observed an inhibition of both Akt phosphorylation and cellular proliferation. They also identified two putative PPREs within the *PTEN* promoter approximately 15 and 13 kb upstream of the ATG site (Figure 2). While this study was significant in demonstrating a potential link between PPAR*γ* and PTEN, it remained correlative.

In 2005, two independent laboratories confirmed Patel’s suspicion that PPAR*γ* induces *PTEN* transcription in a breast cancer setting [21, 22]. We demonstrated that of the four TZDs, only Rosi had the ability to induce *PTEN* transcription and subsequently its protein expression in MCF-7 cells [21]. Furthermore, we showed that stimulation with Rosi induces a PTEN protein that is both protein- and lipid-phosphatase active, as evidenced by decreased phosphorylation of Akt and MAPK concomitant with PTEN expression. Additionally, Rosi treatment induced G1 arrest that paralleled with PTEN expression. By using a Rosi analog, Compound 66, that is incapable of activating PPAR*γ*, we confirmed that Rosi induced PTEN expression via a PPAR*γ*-dependent mechanism in several reporter assays [21].

Additionally, in 2005, Bonofiglio et al. also demonstrated that PPAR*γ* could upregulate *PTEN*’s transcription in a breast cancer setting [22]. After cells were stimulated with Rosi, an increase in PTEN protein was observed as well as an inhibition of Akt phosphorylation and cellular growth. More importantly, they were able to observe for the first time the specific binding of PPAR*γ* to the *PTEN* promoter (−15376 to −15364; Figure 2). Interestingly, this interaction was enhanced by Rosi treatment. Further analysis indicated that PPAR*γ* and estrogen receptor (ER) could bind to the PPRE both independently and simultaneously. The ER’s association with the PPRE inhibited PPAR*γ*’s ability to induce transcription as demonstrated by cotreatment of MCF-7 breast cancer cells with both Rosi and 17ß-estradiol. This cotreatment inhibited the induction of PTEN protein that was observed by Rosi stimulation alone [22]. This is an important observation as it is appealing to postulate that this crosstalk, between PPAR*γ* and ER, may significantly affect breast cancer therapeutics as well as lead the way to the discovery of future novel treatment therapies.

In 2006, Zhang et al. showed that Rosi stimulation of hepatocarcinoma cells results in the upregulation of PTEN and PTEN-dependent inhibition of cell migration [23]. This is significant because PTEN expression is decreased or absent in approximately half of all primary hepatocarcinoma patients. As similarly demonstrated by others, Rosi treatment of hepatocarcinoma cells resulted in an increase in *PTEN* mRNA. They further speculated that there may be three other potential PPREs within the *PTEN* promoter, located at −2874 to −2854, −1615 to −1596, and −1594 to −1574, however, it has not yet been determined if these are functional PPREs. Interestingly, Zhang et al. do not observe an increase in transcriptional activity of the *PTEN* promoter in response to Rosi treatment [23]. We observed similar results when examining the full-length *PTEN* promoter, using a luciferase reporter assay and Rosi stimulation (Teresi, Waite, and Eng; unpublished observations). This may suggest that elements beyond the full-length *PTEN* promoter are required for Rosi-mediated *PTEN* transcription.

These initial studies concretely demonstrated that PPAR*γ* acts as a tumor suppressor in a cancer setting by upregulating *PTEN* transcription. However, these studies were performed solely in breast cancer cell lines, leaving the speculation that these observations are cancer-type dependent. To this end, several groups have studied PPAR*γ*’s ability to regulate PTEN levels in other cancer backgrounds. Lee et al. observed an inhibition of cellular proliferation and Akt phosphorylation in accord with an increase in G1 arrest and PTEN protein expression in A549 lung cancer cells [24]. Subsequently, PPAR*γ* has been shown to upregulate PTEN expression in nonsmall cell lung cancer, neuroblastoma, adrenocortical, pancreatic, heptocarcinoma, and thyroid cell lines [23, 25, 26].

Interestingly, the majority of these studies utilized Rosi as the PPAR*γ* agonist. This may be due to the combination of our initial study, which demonstrated that of the TZDs only Rosi was capable of inducing PTEN expression, and the fact that natural ligands can be difficult to work with in vitro [21]. Despite this, Chen et al. demonstrated that both ciglitazone and 15d-PG-J2 could upregulate PTEN expression in W-2 thyroid cells [27], which raises the possibility that of the TZDs, Rosi stimulation is limited to breast cancer. This remains to be determined.

### 1.4. PPAR*γ* and PTEN in vivo

Despite the growing amount of in vitro data supporting the role of PPAR*γ* as a tumor suppressor, only a small number of cancers have had their PPAR*γ* status characterized in vivo and there are very few studies of clinical PPAR*γ* agonist treatment. Nonetheless, current studies provide some essential and encouraging information. One of the first studies to analyze *PPAR*γ** status in an in vivo cancer setting examined 55 unrelated sporadic colon cancer samples and revealed 4 *PPAR*γ** mutations [28]. Moreover, these mutations produced an inactive PPAR*γ* protein. This study demonstrated that PPAR*γ* can act as a tumor suppressor in vivo and when its normal activity is altered it can lead to cancer development [29]. Subsequent studies have confirmed these results showing the reduction of PPAR*γ* expression in both acrometaly [30] and ulcerative colitis [31], two predisposing conditions of colon cancer. In contrast to these studies, Ikezoe et al. did not observe any *PPAR*γ** alterations in their colon cancer study; however they limited their study to only exons 3 and 5 of *PPAR*γ** [32]. These studies indicate that PPAR*γ* is indeed a tumor suppressor in the colon cancer setting; however none of these studies tested if the TZDs could effect the cancer’s progression.

To date, the majority of studies correlating PPAR*γ* with PTEN have been performed in vitro and these studies suggest that PPAR*γ* agonists may be beneficial to PTEN in vivo. Moreover, in vitro data suggest that PPAR*γ* agonists have the potential to be highly effective *PTEN* transcriptional inducers for patients who have one of the following: a hemizygous deletion, a germline nucleotide alteration within the promoter, and potentially in the circumstance, where a *PTEN* mutation is not identified but a decrease in protein expression is observed.

Despite the potential beneficial effects of TZD treatment, in particular Rosi, one must be aware that the use of these medications may lead to more harm than good. For example, treatment of patients with germline intragenic *PTEN* mutations or those with neoplasias containing somatic intragenic mutations may see a raise of mutant, inactive protein. Recently, PTEN has been shown to induce gain-of-function p53 protein suggesting that TZD treatment in this setting may subsequently induce mutant, nonbeneficial p53 protein. Additionally, our work and others have suggested that not all of TZDs signal through the same pathways, at least in cell culture conditions [21]. Rosi is the only TZD that is known to increase PTEN in breast cancer lines, which indicates that each TZD may lead to its own individual side effects. Indeed in 2000, troglitazone (Rezulin) was pulled off of the market due to liver toxicity. Interestingly, to date, this has not been observed with other TZDs [33]. A recent study demonstrated that Rosi (Avandia) increases the risk of heart complications, specifically heart attacks; however these results have yet to be replicated [34]. This indicates that the significance of Rosi treatment on cardiac function needs to be examined further. Indeed, in this first study, important results, which came to the opposite conclusions, were not included in the meta-analysis. In spite of this, a deeper understanding of the signaling mechanisms behind these side effects should open the door to both new avenues of cancer treatment and personalized health care, allowing physicians to properly weigh the benefits against the known side effects prior to prescribing such a treatment.

Drug-drug interactions are another aspect that physicians will need to be aware of. Bonofiglio’s PPAR*γ*-ER-PTEN results are significant in the context of breast cancer and hormone therapies [22]. Their data suggest that women treated with hormones, either through birth control or hormonal therapies, may not benefit from cotreatment with a PPAR*γ* agonist. This further suggests that hormone treatment may actually be detrimental by inhibiting naturally occurring *PTEN* transcription.

### 1.5. The translation of PPAR*γ* and PTEN into the clinic

A recent study has suggested that Rosi treatment could be beneficial to patients with Gefitinib-resistant lung cancer [24], a cancer which is typically correlated with the loss of PTEN protein. Lee et al. have shown that in the human lung cancer cell line, A549, the combined treatment of Rosi and Gefitinib was more beneficial than Gefitinib treatment alone [24]. Taken together, these data provide support that the upregulation of PTEN levels with Rosi treatment may reverse the Gefitinib resistance in these patients. Such a treatment could have the potential to be advantageous to patients with both sporadic and familial cancer.

PPAR*γ* status is only now beginning to be examined in the in vivo cancer setting, however the TZDs have been used in a variety of clinical trials, although not directly related to PPAR*γ* activation. Seemingly, out of the ordinary, polycystic ovary syndrome (PCOS) is the most commonly studied syndrome with regards to the effects of TZD treatment [35]. While there is still much debate on what treatment is best for these patients, the majority believe that Rosi treatment is beneficial. Studies have demonstrated that Rosi treatment raises insulin and androgen levels in the obese PCOS population, thereby inhibiting tumor progression. Furthermore, Yee et al. recently performed a pilot study in women with breast cancer to determine if Rosi treatment would be beneficial. Thirty-eight women with early stage breast cancer were treated with Rosi for 2–6 weeks with tumor growth inhibition or progression as an end point [36]. The data indicate that short-term Rosi therapy in early-stage breast cancer patients has both local and systemic effects on PPAR*γ* signaling. Both of these studies suggest that Rosi may be used clinically to benefit cancer patients.

### 1.6. PPAR*γ* and PTEN’s future

The culmination of these data strongly suggests that Rosi stimulation may be advantageous to the cancer patient. However, lacking in many of these studies is the role of PTEN. To date, in vitro data has demonstrated a connection between PPAR*γ* and PTEN, yet no in vivo study has concretely confirmed these results. The results obtained from these studies would concretely determine if Rosi treatment is advantageous for cancer patients by upregulating PTEN expression through PPAR*γ*.

While clinical trials are necessary to determine if Rosi treatment is truly beneficial for cancer patients and which patients it is most advantageous for, much remains to be learned at the molecular level. The relevance of the putative PPRE in the *PTEN* promoter identified by Bonofiglio et al. remains to be determined (Figure 2) [22]. This PPRE is located a long distance from the ATG site, thus making it unclear if this site is functional in regulating PTEN expression. It will be interesting to find out the role of this unique site.

While evidence suggests that TZDs induce PTEN expression through PPAR*γ*, further studies are warranted to determine the exact mechanism of action. Evidence by our group suggests that PPAR*γ* may regulate PTEN expression through both transcriptional-dependent and -independent mechanisms [37]. While this may add to the complexity of the role of PPAR*γ*, with regards to PTEN, it may also provide other areas for therapeutic advances. Interestingly, while studying the ability of statins to induce PTEN expression, we observed that statins increase *PTEN* transcription via an unknown PPAR*γ*-mediated mechanism [37]. Retrospectively, we observed a similar response with Rosi stimulation indicating that PPAR*γ* is necessary; however its transcriptional activity is not. These results suggest that PPAR*γ* may induce *PTEN* transcription through an unknown mechanism and an unrecognized transcription factor; however this remains to be determined.

## 2. CONCLUSION

In recent years, there has been a growing accumulation of data implicating the importance of both PPAR*γ* and PTEN in cancer prevention, development, and treatment. In vitro data has demonstrated that PPAR*γ* agonists can induce functional PTEN protein that controls cellular growth. In vivo data has suggested that *PPAR*γ** genetic alteration can lead to cancer development, while its agonists can inhibit tumor progression. Despite this progress, we are only beginning to determine the roles of these two proteins and their complex interactions. Undoubtedly, future studies will clarify the PPAR*γ*-*PTEN* connection providing a variety of targets that may lead to novel therapeutic treatments for cancer patients.
